# The Biological Activity of Natural Alkaloids against Herbivores, Cancerous Cells and Pathogens

**DOI:** 10.3390/toxins11110656

**Published:** 2019-11-11

**Authors:** Amin Thawabteh, Salma Juma, Mariam Bader, Donia Karaman, Laura Scrano, Sabino A. Bufo, Rafik Karaman

**Affiliations:** 1 Samih Darwazah Institute for Pharmaceutical Industries, Faculty of Pharmacy Nursing and Health Professions, Birzeit University, Bir Zeit 71939, Palestine; 2 Pharmaceutical Sciences Department, Faculty of Pharmacy, Al-Quds University, Jerusalem 20002, Palestine; salmajuma_1989@yahoo.com (S.J.); mariam407@hotmail.com (M.B.); 3 Department of Science, University of Basilicata, 85100 Potenza, Italy; kdonia65@yahoo.com (D.K.); sabino.bufo@unibas.it (S.A.B.); 4 Department of European Cultures (DICEM), University of Basilicata, 75100 Matera, Italy; laura.scrano@unibas.it

**Keywords:** alkaloids, natural sources, anticancer, antibacterial, antiviral, antifungal

## Abstract

The growing incidence of microorganisms that resist antimicrobials is a constant concern for the scientific community, while the development of new antimicrobials from new chemical entities has become more and more expensive, time-consuming, and exacerbated by emerging drug-resistant strains. In this regard, many scientists are conducting research on plants aiming to discover possible antimicrobial compounds. The secondary metabolites contained in plants are a source of chemical entities having pharmacological activities and intended to be used for the treatment of different diseases. These chemical entities have the potential to be used as an effective antioxidant, antimutagenic, anticarcinogenic and antimicrobial agents. Among these pharmacologically active entities are the alkaloids which are classified into a number of classes, including pyrrolizidines, pyrrolidines, quinolizidines, indoles, tropanes, piperidines, purines, imidazoles, and isoquinolines. Alkaloids that have antioxidant properties are capable of preventing a variety of degenerative diseases through capturing free radicals, or through binding to catalysts involved indifferent oxidation processes occurring within the human body. Furthermore, these entities are capable of inhibiting the activity of bacteria, fungi, protozoan and etc. The unique properties of these secondary metabolites are the main reason for their utilization by the pharmaceutical companies for the treatment of different diseases. Generally, these alkaloids are extracted from plants, animals and fungi. Penicillin is the most famous natural drug discovery deriving from fungus. Similarly, marines have been used as a source for thousands of bioactive marine natural products. In this review, we cover the medical use of natural alkaloids isolated from a variety of plants and utilized by humans as antibacterial, antiviral, antifungal and anticancer agents. An example for such alkaloids is berberine, an isoquinoline alkaloid, found in roots and stem-bark of *Berberis asculin* P. Renault plant and used to kill a variety of microorganisms.

## 1. Introduction

Ample research has been conducted on natural products in order to obtain new antimicrobial agents to compensate for the increasingly microbial resistance. In fact, the problem with the currently used antiviral drugs is the development of resistance by the microorganism.

Many traditionally used plants for viral infections have been studied. Extracted compounds, including terpenes (e.g., mono-, di- and tri-), flavonoids, phenols and polyphenols have been found to be active against HSV virus [1,2]. Flavonoids extracted from plants are utilized by people for their health benefits, and have been shown to have viral activity against HCMV [3]. Fourteen new alkaloids were isolated from *Cladosporium* species (spp.) PJX-41 fungi and showed inhibitory activity against influenza *virus A (H1N1)* [4].

### The Biological Activities of Alkaloids

Alkaloids are grouped into several classes. This classification is based on their heterocyclic ring system and biosynthetic precursor. They include tropanes, pyrrolidines, isoquinoline purines, imidazoles, quinolizidines, indoles, piperidines and pyrrolizidines. There is a great interest in the chemical nature of these alkaloids and their biosynthetic precursors. Alkaloids have been extensively researched because of their biological activity and medicinal uses. Serotonin and other related compounds are belonging to the commonly used insole alkaloids. It is estimated that about 2000 compounds are classified as indole alkaloids. They include vinblastine, strychnine, ajmaline, vincamine, vincristine and ajmalicine, which are among the most researched members, due to their pharmacological activities. For example, vincristine and vinblastine, named spindle poison, are generally utilized as anticancer agents [5]. Convolvulaceae, Erythroxylaceae and Solanaceae families include the pharmacologically active tropane alkaloids which have an 8-azabicyclo octane moiety derived from ornithine [5,6]. Hyoscyamine, cocaine, scopolamine and atropine alkaloids are the most known members of this group and possess a variety of pharmacological effects. Quinoline and isoquinoline known as benzopyridines are heterocyclic entities containing fused benzene, and pyridine rings have many medical uses [6]. Quinine, a quinoline alkaloid isolated from *Cinchona ledgeriana* (Howard) and *Calendula officinalis* L. was proved to be poisonous to *Plasmodium vivax* and organisms with single cell or *Protozoans* that cause malaria. Other members of the quinine alkaloids include cinchonidin, folipdine, camptothecin, chinidin, dihydroquinine, echinopsine and homocamptothecin [5,7]. These chemical entities have demonstrated significant pharmacological effects, such as anticonvulsant, analgesic, antifungal, anthelmintic, anti-inflammatory, antimalarial, anti-bacterial andcardiotonic [7]. Other important alkaloids are those derived from isoquinoline, a quinoline isomer, which are classified into various classes, based on the addition of certain groups: Phthalide isoquinolines, simple isoquinolines and benzylisoquinolines. Among the well-known alkaloids belong to this category are morphine (analgesic and narcotic drug), codeine (cough suppressant), narcotines, protopines, and thebaine [8]. In addition, this class of alkaloids has demonstrated various pharmacological activities, such as antitumor, antihyperglycemic and antibacterial [6]. Among the most important alkaloids from the purine class (xanthenes) are theophylline, aminophyline and caffeine. This class of alkaloids possesses a variety of pharmacological activities, including anti-inflammatory, antioxidant, antidiabetic, anti-obesity and anti-hyperlipidemic [9]. On the other hand, the alkaloids derived from piperidine are generally obtained from *Piper nigrum* L. and *Conium maculatum* L. plants. It is estimated that 700 members of this class have been researched. These alkaloids possess a saturated heterocyclic ring (piperidine nucleus) and are familiar with their toxicity. They have many pharmacological activities which include anticancer, antibacterial, antidepressant, herbicidal, anti-histaminic, central nervous system stimulant, insecticidal and fungicidal [10,11]. The famous poison of hemlock known as *Conium maculatum* presents in the piperidine alkaloids. Members of the piperidine alkaloids include lobeline, coniine and cynapine. The pyridine alkaloids have a quite similar chemical structure to that of piperidine alkaloids except the unsaturated bonds exist in their heterocyclic nucleus. Anatabin, anatabine, anabasin, epibatidine and nicotine are some members of the pyridine alkaloids [12]. Imidazole alkaloids are compounds containing an imidazole ring in their chemical structure and are derived from L-histidine. The most known member of this class is pilocarpine which is obtained from *Pilocarpus cearensis* Rizziniand is used as a drug in ophthalmic preparations to treat glaucoma [13]. The pyrrolizidine alkaloids, containing a necine base, are present only in plants, such as Leguminosae, Convolvulaceae, Boraginaceae, Compositae, Poaceae and Orchidaceae. Among the most known members of this class are heliotrine, echinatine, senecionine and clivorine which are biosynthesized by the plants for protection from herbivores. These are hepatotoxic causing several diseases, such as liver cancer. Due to their glycosidase inhibition activity, they are used to treat diabetes and cancer [14]. Pyrrolidine alkaloids are compounds composed of aza five membered rings that are derived from ornithine and lysine. Hygrine, cuscohygrine and putrescine are some members of this class. Biological studies conducted on this class have revealed significant antifungal, antitubercular and antibacterial activities among a large number of these compounds [15]. 

Quinolizidine alkaloids contain two fused 6-membered rings that share nitrogen and derived from the genus Lupinus and are known as lupine alkaloids. Among the members of this class are lupinine and lupanine cytisine and sparteine. The last two members are the most distributed quinolizidine alkaloids and are characterized by their antimicrobial activities [16]. 

Bacterial infections are considered as a major health problem worldwide. Moreover, they are increasing, due to multidrug resistance, which subsequently causes mortality and morbidity. Therefore, new antibacterial remedies are needed, and the plants represent a wide source for novel natural compounds [17]. Three alkaloids solanine, solasodine and *B*-solamarine have been extracted from *Solanum dulcamara* L. (Solanaceae), commonly known as bittersweet plant, and have demonstrated significant antibacterial activity against *Staphycoccus aureus* [17]. Bis-indole alkaloids were obtained from *marine invertebrates* and showed antibacterial activity against *S. aureus*, including MRSA (methicillin resistance *Staphycoccusaureus*) [18]. Berberine and hydrastine alkaloids were extracted from Goldenseal (*Hydrastis canadensis* L., *Ranunculaceae*) and have demonstrated a potent antibacterial activity mostly against *Streptococcus pyogenes* and *Staphycoccus aureus* [19]. Cocsoline alkaloid was isolated from *Epinetrumvillosum* (Exell), it has a wide antibacterial activity; inhibits *Shigella strains*, *Campylobacter jejuni* and *Campylobacter coli* [20].

Antifungal agents to treat fungal infections have serious side effects and developed fungal resistance, hence, there is a pressing need to look for new and novel antifungal agents. Alkaloids extracted from the leaves of *Ruta graveolens* L. were shown to possess fungitoxic activity [21]. Tomadini Glycoalkaloids have been extracted from tomato and proved to have antifungal activity [22]. Quinoline alkaloids and flavonoids extracted from *WaltheriaIndica* L. Roots were approved to have antifungal activity against *Candida albicans* [23]. 

Cancer is second in the list of diseases causing death worldwide. Phytochemicals represent a source for anticancer agents, due to their low toxicity and high effectiveness. Hersutin alkaloid is a major alkaloid found in *Uncaria* genus; hersutin was found to cause apoptosis in HER2-positive and the p53-mutated breast cancer cells [24]. Oxymatrine, a natural alkaloid extracted from *Sophora chrysophylla* (Salisb.) roots, was found to have anticancer activity in human cervical cancer Hela cells, due to its cytotoxic effects and apoptosis [25].

Herein, we report a comprehensive review on the medical use of some natural alkaloids, such as antibacterial, antiviral, antifungal and anticancer agents.

## 2. Natural Alkaloid Used to Control Agricultural Pests (Herbivores)

Glycoalkaloids extracted from Potato leaves were demonstrated to exert negative effects on the hatching success of *Spodoptera exigua* eggs, and on the heart contractile activity of three beetle species *Zophobas atratus*, *Tenebrio molitor*, and *Leptinotarsa decemlineata* [26].

Similar effects were shown on *Zophobas atratus* F. and *Tenebrio molitor* L. by commercial glycoalkaloids (solamargine, solasonine, α-chaconine, α-solanine, α-tomatine) and by aqueous extracts from *Solanum etuberosum* L., *Lycopersicon esculentum* Mill., and *Solanum nigrum* L. [27]. Furthermore, Potato leaf extracts and commercial α-solanine were proved to influence the life history parameters and antioxidative enzyme activities in the midgut and fat body of *Galleria mellonella* L. [28]. Additionally, *Solanum tuberosum* L, *Lycopersicon esculentum* Mill.,and *Solanum nigrum* L. Leaf extracts and single pure glycoalkaloids have been demonstrated to affect the development and reproduction of *Drosophila melanogaster* [29,30].

## 3. Natural Alkaloid Used as Anticancer Agents

**Figure 1:**
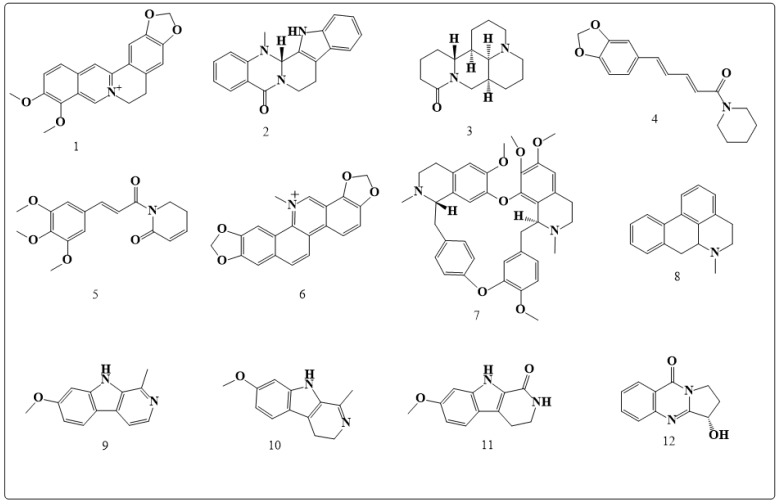
Chemical structures ofanticancer natural alkaloids: Berberine (**1**), evodiamine (**2**), matrine (**3**), piperine (**4**), piplartine (**5**), sanguinarine (**6**), tetrandrine (**7**), aporphine (**8**), harmine (**9**), harmaline (**10**), harmalacidine (**11**) and vasicinone (1**2**).

Alkaloids are the most biologically active compounds found in natural herbs and the source of some important drugs currently marketed. These include some anticancer agents, such as camptothecin (CPT) and vinblastine. The cytotoxicity and mechanisms of action for the following derived alkaloids, berberine, evodiamine, matrine, piperine, piplartine, sanguinarine, tetrandrine, aporphine, harmine, harmaline, harmalacidine and vasicinone, (**1**–**12,** respectively, in Figure 1), is our main focus in this section, since they are believed to have fewer side effects and lower resistance compared to other chemotherapeutic agents.

Berberine (**1** in Figure 1) is an isoquinoline derivative extract from *Coptis chinensis* Franch, Berberidaceae.This secondary metabolite possesses a variety of pharmacological effects, which include antibacterial, antidiabetes, anti-inflammatory and antiulcer ones. In addition, Also, it had been found to be beneficial for the cardiovascular system [19,20,22,23]. It has been demonstrated that *Coptis chinensis* Franch, the plant in which berberine present, has an inhibitory effect on proliferation of breast and liver cancer cells. The anticarcinogenic activity of berberine was studied in FaDu cells, which are human pharyngeal squamous carcinoma cells; berberine was found to have a cytotoxic effect and has decreased the viability of these cells in a concentration-dependent manner [31]. In vitro and in vivo experiments on berberine have demonstrated anticancer activity by causing cell cycle arrest at the G1 or G2/M phases and tumor cell apoptosis [24,32]. In addition, berberine was found to cause endoplasmic reticulum stress and autophagy, which had resulted in inhibition of tumor cell metastasis and invasion [2,33,34,35,36].

Besides its apoptotic effects, berberine was found to reduce angiogenesis by reducing VEGF expression. Also, it resulted in decreased cancer cell migration [31]. The anticancer activity of berberine was studied in the human promonocytic U937 and murine melanoma B16 cell line; cytotoxic activity was found to be concentration-dependent. Intraperitoneal administration of berberine in mice had caused a reduction of 5 to 10 kg of tumor weight after a treatment of 16 days. This reduction in tumor weight was found to be time and concentration-dependent [33].

**Figure 2:**
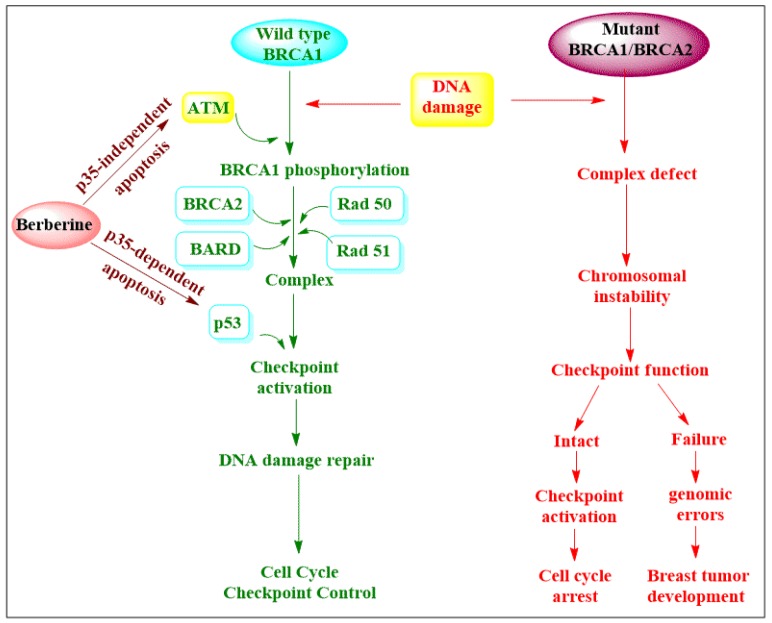
Anticancer mechanism of action.

Berberine binds to DNA or RNA to form the corresponding complexes [37,38]. Berberine also inhibits a number of enzymes, including cyclooxygenase-2 (COX-2), *N*-acetyltransferase (NAT) and tolemerase [24]. It has many effects on tumor cells, which include cyclin-dependent kinase (CDK) regulation [24,37] and expression regulation of *B*-cell lymphoma 2 (Bcl2) proteins (Bax, Bcl-2, Bcl-xL) [24,33,37] and caspases [33,37]. Further, berberine has an inhibition activity on nuclear factor κ-light-chain enhancer of activated B cells (NF-κB) and activation on the synthesis of intracellular ROS [24,33]. It is worth noting that berberine activity is selective for cancer cells [24]. Studies have shown that berberine affects tumor cell progression by the inhibition of focal adhesion kinase (FAK), urokinase, matrix metalloproteinase 9 (MMP-9), NFκB and matrix metalloproteinase2 (MMP-2) [2,39]. In addition, it reduced Rho kinase-mediated Ezrin phosphorylation [36], inhibited COX-2 synthesis, prostaglandin receptors and prostaglandin E [40]. Other activities of berberine include inhibition of the synthesis of hypoxia-inducible factor 1 (HIF-1), proinflammatory mediators and vascular endothelial growth factor (VEGF) [41,42]. Berberine also activates P53 gene, which results in apoptosis and cell cycle arrest. It has been demonstrated that berberine also caused apoptosis by mitochondrial-dependent pathway and interactions with DNA, as shown in Figure 2 [32]. 

Evodiamine (**2** in Figure 1), a quinolone alkaloid, isolated from the Chinese plant *Evodia rutaecarpa* has a variety of pharmacological activities against obesity, allergy, inflammation, anxiety, nociception, cancer, thermoregulation. In addition, it is a vessel-relaxing activator and an excellent protector of myocardial ischemia-reperfusion injury [43,44,45,46]. It causes cell cycle arrest, apoptosis, inhibits the angiogenesis, invasion, and metastasis in different cancer cells at G2/M phase in most cancer cell lines [47,48,49,50,51]. In vitro studies have shown evodiamine to be quite active against cancer cell progression at micromolar-nanomolar concentrations [52,53]. Additionally, evodiamine stimulates autophagy, which prolongs survival [54]. Evodiamine is selective for tumor cells and less toxic to normal human cells, such as human peripheral blood mononuclear cells. Studies have shown that it inhibits the proliferation of Adriamycin resistant human breast cancer NCI/ADR-RES cells, both in vitro and in vivo, and was found to be active when administered orally [49]. Moreover, it was found that the administration of 10 mg/kg evodiamine from the 6th day after tumor injection into mice reduced lung metastasis without affecting the mice weight [47]. Studies showed that evodiamine inhibits TopI enzyme, forms a DNA covalent complex with a close concentration, 2.4 µM and 4.8 µM, to that of CPT, and induces DNA damage [55,56,57]. It exhibited G2/M phase arrest [47,49,58] and not S phase arrest, which is not consistent with the mechanism of TopI inhibitors, such as CPT, which indicates that evodiamine has targeted other than TopI, such as tubulin polymerization [58]. Evodiamine was found to induce intracellular ROS production and cause mitochondrial depolarization [59]. Mitochondria apoptosis is caused by the generation of ROS and nitric oxide [54]. Evodiamine was also found to trigger caspases dependent and caspase-independent apoptosis, downregulates Bcl-2 expression, and upregulates Bax expression in some cancer cells [50,52]. The phosphatidylinositol 3kinase/Akt/caspase and Fas ligand (Fas-L)/NF-κB and ubiquitin-proteasome pathway are considered the route in which evodiamine causes induction to cells death [53].

Matrine (**3** in Figure 1) is an alkaloid isolated from *Sophora flavescens* Aitonplants [60]. It possesses a variety of pharmacological activities which include diuretic, choleretic, hepatoprotective, nephroprotective, antibacterial, antiviral, anti-inflammatory, antiasthmatic, antiarrhythmic, antiobesity, cardioprotective and anticancer [61,62,63,64,65,66,67]. In the treatment of cancer matrine is used in high doses, but it has not shown any major effects on normal cells viability [61,62,63,64,65,66,67,68,69,70,71]. G1 cell cycle arrests mediation and apoptosis is the mechanism, by which matrine exerts its inhibition effects on the proliferation of different cancer cells [68,69,71,72,73]. Matrine triggers apoptosis and autophagy in cancer cells, such as hepatoma G2 cells and SGC7901 cells. Matrine also induces the differentiation of K562 tumor cells and has antiangiogenesis activity [74]. The in vivo anticancer effects of matrine has been tested in H22 cells, MNNG/HOS cells, 4T1 cells and BxPC-3 cells in BALB/c mice [70,71,74,75]. It was found that matrine at 50 mg/kg or 100 mg/kg inhibits MNNG/HOS *Xenograft* growth, and reduces the pancreatic tumor size compared to controls at similar doses [71]. Targets of matrine are still under study until now, but it was found that it affects many proteins involved in cell proliferation and apoptosis, such as E2F-1, Bax, Bcl-2, Fas, and Fas-L [68,70,71,72,73,76]. It also inhibits cancer cell progression by the inhibition of MMP-2 and MMP-9 expression and modulation of the NF-κB signaling pathway [77,78,79]. 

Piperine (**4** in Figure 1) is a piperidine alkaloid found in *Piper nigrum* and *Piper longum* [80]. It exhibits antioxidant, antidiarrheal, anti-inflammatory, anticonvulsant, anticancer and antihyperlipidemic properties in addition to being a trigger for bile secretion production [65,81] and suppressant to the CNS system [82,83]. An administration of 50 mg/kg or 100 mg/kg of piperine daily for a week significantly reduced the size of solid tumor in mice transplanted with sarcoma 180 cells. Recently, a study has revealed that piperine has managed to inhibit the progression of breast cancer in a selective manner [84]. It has been shown that this secondary metabolite triggered cell cycle arrest in G2/M phase and apoptosis in 4T1 cells [85,86]. Piperine in a concentration of 200 μM/kg is also active against lung cancer metastasis induced by B16F-10 melanoma cells in mice [87] and causes suppression of phorbol-12-myristate-13acetate (PMA), which induce tumor cell invasion [88]. Piperine inhibits NF-κB, c-Fos, cAMP response element-binding (CREB) and activated transcription factor 2 (ATF-2) [89]. It suppresses PMA-induced MMP-9 expression through the inhibition of PKCα/extracellular signal-regulated kinase (ERK) ½ and reduction of NF-κB/AP-1activation [88]. Piperine also inhibits P-glycoprotein (P-gp) and CYP3A4 activity, which affects drug metabolism and also re-sensitizes multidrug resistant (MDR) cancer cells [90,91]. Piperine increases the effect of docetaxel without inducing more side effects on the treated mice by inhibiting CYP3A4 which is the main metabolizing enzymes of docetaxel [92].

Piplartine, the less common name for piperlongumine, (**5** in Figure 1) is an amide-alkaloid obtained from *Piper longum* L.It has resulted in tumor growth inhibition in *Sarcoma180* cells transplanted in mice. This antitumor activity was due to its antiproliferative effect [93].

Sanguinarine (**6** in Figure 1) is a benzophenanthridine obtained from *Sanguinaria canadensis L*. and *Chelidoniummajus L*. [94,95]. It is active against bacterial, fungal and schistosomal infections, antiplatelet, and anti-inflammatory properties [96,97,98], it is also utilized for schistosomiasis control and cancer treatment. In vitro studies showed that it presents anticancer effects at concentrations less than ten micromoles. Sanguinarine triggers cell cycle arrest at different phases of apoptosis in many tumor cells [99,100,101,102,103,104]. It also is active against angiogenesis [105,106,107]. The administration of COX-2 inhibitors and sanguinarine has been recommended for prostate cancer treatment. Sanguinarine can also be used for the treatment of conditions caused by ultraviolet exposure, such as skin cancer [108]. The mechanism of its anticancer activity could be its interactions with glutathione (GSH). These interactions decrease cellular GSH and increase ROS generation [102,109]. Sanguinarine-induced ROS production and cytotoxicity can be blocked by pretreatment with N-acetyl cysteine or catalase. This mode of action is similar to that of TopII inhibitor salvicine [110,111]. Sanguinarine is considered a potent inhibitor of MKP-1 (mitogen-activated protein kinase phosphatase 1) [112]. It also interferes with microtubule assembling [113] and the nucleocytoplasmic trafficking of cyclin D1and TopII, and causes DNA damage leading to anticancer activity. Sanguinarine suppresses NF-κB activation triggered by TNF, interleukin-1, okadaic acid, and phorbol ester, but not that induced by hydrogen peroxide or ceramide [114]. It also inhibits the signal transducer and activator of transcription 3 activation (STAT-3) [115]; downrgulates CDKs, cyclins, MMP-9 and MMP-2 [106,109]; upregulates p21, p27 [104,107] and the phosphorylation of p53 [99]; alters the members of the Bcl-2 family, including Bax, Bid, Bak Bcl-2, and Bcl-xL; activates caspases [101,102,103]; and upregulates death receptor 5 (DR-5) [101].

Tetrandrine (**7** in Figure 1) is a bisbenzylisoquinoline alkaloid extracted from *Stephania tetrandra* S.MooreIt is used as an immune modulator, antihepatofibrogenetic, antiarrhythmic, anti-inflammatory, antiportal hypertension, anticancer and neuroprotective agent [116]. It is a potent anticancer agent. Tetrandrine triggers different phases of cell cycle arrest depending on the cancer cell type, and also induces apoptosis in many human cancer cells, including leukemia, colon, bladder, lung and hepatoma [117,118]. In vivo experiments demonstrated that the survival of mice subcutaneously injected with CT-26 cells was prolonged after daily treatment with tetrandrine [119]. Tetrandrine suppresses the expression of VEGF in glioma cells, has an anticancer effect on ECV304 human umbilical vein endothelial cells, and aniangiogenesis effects [120]. Tetrandrine had no acute toxicity were noticed [121]. Hence, tetrandrine has a great promise as an MDR modulator for the treatment of P-gp-mediated MDR cancers. Tetrandrine could be used in combination with 5-fluorouracil and cisplatin drugs [122,123]. If combined with tamoxifen it increases the efficacy by inhibiting phosphoinositide-dependent kinase 1 [124]. It also increases the radio-sensitivity of many cancer cells by affecting the radiation-induced cell cycle arrest and interfering with the cell cycle. So tetrandrine can be used in combination with cancer chemotherapy or radiotherapy. Activation of glycogen synthase kinase 3β (GSK-3β), generation of ROS, activation of p38 mitogen-activated protein kinase (p38 MAPK), and inhibition of Wnt/betacaten in signaling might cause the anticancer activity of tetrandrine [119,125]. Tetrandrine up-regulates p53, p21, p27, and Fas; down-regulates Akt phosphorylation, cyclins, and CDKs and activates caspases [120,125,126,127].

Aporphine alkaloids (**8** in Figure 1) are extracted from the aerial part of *Pseuduvariasetosa*. These alkaloids demonstrated moderate antitumor activity against lung and breast cancer cells, in addition to their high antitumor activity against *epidermoid carcinoma* (KB) and breast cancer [36]. Mohamed et al. have studied *Magnolia grandiflora* L., characterized the isolated aporphine alkaloids and researched their cytotoxic activity. They found that the magnoflorine and lanuginosine alkaloids have cytotoxic activity against liver carcinoma cell lines [2].

Apomorphine alkaloids were isolated from *Nelumbo nucifera* Gaertn leaves and were demonstrated to have antioxidant and antipoliferative activities [41]. 

*Peganum. Harmala* has been used traditionally for cancer therapy, harmine (**9** in Figure 1) and harmaline (**10** in Figure 1) are the major alkaloids found in this plant in ratios of 4.3% and 5.6%, respectively [128]. Four alkaloids; harmine, (**9** in Figure 1), harmaline (**10** in Figure 1), harmalacidine (**11** in Figure 1) and vasicinone (**12** in Figure 1) were isolated from plant seeds and have shown a significant cytotoxic activity [128]. The mechanism of action of P. *harmala* seeds has been investigated by Sobhani et al.; it was found that these alkaloids inhibit topoisomerase **1**, resulting in antiproliferation of cancer cells [129]. 

A concise summary of the anticancer activities of alkaloids **1**–**12** is depicted in Table 1.

## 4. Antibacterial Activities

Anti-bacterial agent is a chemical entity that has the potential to kill or inhibit the production of bacteria. Since antiquity several nations have been using different plants for healing and treating several kinds of diseases, like using, *Ayurveda*, in providing many medicines from the *Neem tree*, *Azadirachta indica* A.Juss.in India, and *Valerian (Valeriana officinalis)*, the medicinal plant endogenous to Europe and Asia and widely introduced in North America [37,39,130]. However, at the beginning of the 1980s this trend has declined, and the sights of researchers were turned into synthesizing new compounds. More recently the interest in using natural products has been renewed, due to the huge increase in antibiotics resistance, the limited availability of new synthetic antibacterials [37], and the discovery of many new natural products [38]. Finding new anti-infective agents is a necessity. Even with the careful use of antibiotics, each antibiotic has a limited life span [37]. Nothing can rule out the need for antibiotics, although biologics can sometimes be used in several cases, but they are usually accompanied by many limitations [39,40].

Several studies have demonstrated that much plant extracts containingalkaloids, flavonoids, phenolics and other compounds have significant antibacterial activity. However, in this section, we are specifically concerned with alkaloids as antibacterial agents.

Alkaloids have a reputation of being a natural curse and blessing [131]. They are a wide-range and diverse group of natural compounds that exist in plants, animals, bacteria, and fungi. The only thing they have in common is the occurrence of a basic nitrogen [132], which can bea primary, secondary or tertiary amine. Currently, there are more than 18,000 discovered alkaloids [133]. The unique bioactivity of alkaloids is attributed to the presence of nitrogen, that capable of accepting a proton, and one or more amine donating hydrogen atoms, which is usually accompanied by proton-accepting and -donating functional [127].

Alkaloids have played a very important role in developing new antibacterial agents. Many interesting examples include the synthesis of quinolones from quinine, derivatization of azomycin to afford metronidazole, and alteration of the quinoline scaffold to furnish bedaquiline. In other antibacterial agents, alkaloids are utilized as scaffold substructures as seen with linezolid and trimethoprim [134,135,136].

### 4.1. Antibacterial Indole Alkaloids 

**Figure 3:**
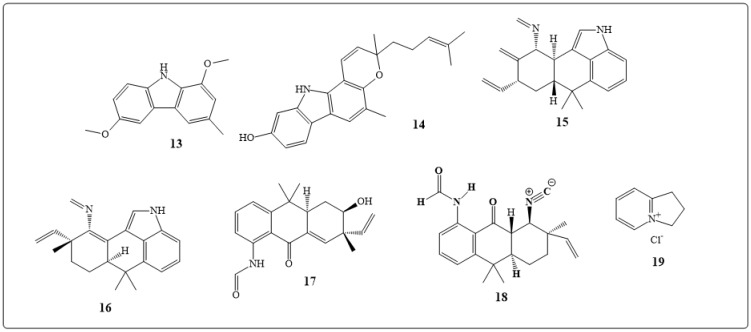
Chemical structures of natural antibacterial alkaloids: Clausenine (**13**), *(R)*-Mahanine (**14**), hapalindole X (**15**), deschlorohapalindole I (**16**), 13-hydroxy dechlorofontonamide **(17**), hapalonamide H (**18**) and 2,3-dihydro-1 H-indolizinium chloride (**19**).

Clausenine (**13** in Figure 3) extracted from the stem bark of *Clausena anisate* (Willd.) were shown to have antibacterial and antifungal activities [137]. *I*-Mahanine indole alkaloid (**14** in Figure 3) obtained from *Micromelum minutum* Wight and Arn. has demonstrated antimicrobial activity against *Bacillus cereus* (MIC100 values of 6.25 μg/mL) and *Staphylococcus aureus* (MIC100 values of 12.5 μg/mL) [138].

Hapalindole alkaloids, hapalindole X (**15** in Figure 3), deschlorohapalindole I (**16** in Figure 3), and 13-hydroxy dechlorofontonamide (**17** in Figure 3) and hapalindoles A, C, G, H, I, J, and U, hapalonamide H, anhydrohapaloxindole A, and fischerindole L were obtained from *cyanobacteria sp*. Results demonstrated that **15** and **18** possess a very strong activity against both *Mycobacterium tuberculosis* and *Candida albicans* with MIC values in the range of 0.6 to 2.5 μM [139].

The indolizdine alkaloid 2, 3-dihydro-1H-indolizinium chloride (**19** in Figure 3) isolated from *Prosopisglandulosa* Torr. *var. glandulosa* was found to be a strong antifungal and antibacterial agent against *Cryptococcus neoformans, Aspergillus fumigatus* with IC50 values of 0.4 and 3.0 μg/mL, respectively, and antibacterial activity against methicillin-resistant *Staphylococcus aureus* and *Mycobacterium* with IC50 values of 0.35 and 0.9 μg/mL respectively [140]. 

**Figure 4:**
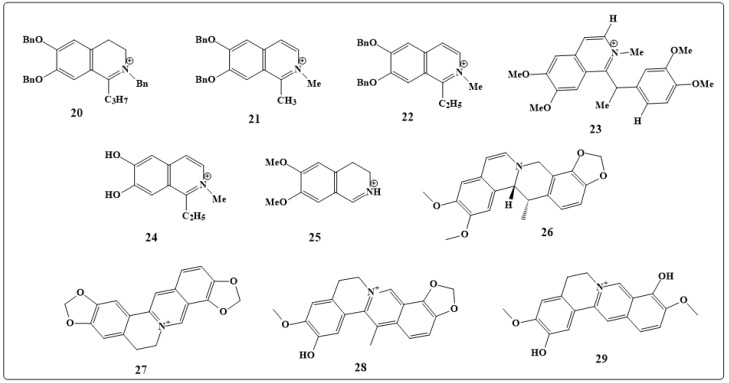
Chemical structures of isoquinolines alkaloids (antimicrobial, antimalarial, cytotoxic, and anti-HIV agents, **20**–**25**), 2-benzyl-6,7-bis(benzyloxy)-1-propyl-3,4-dihydroisoquinolin-2-ium (**20**), 6,7-bis(benzyloxy)-1,2-dimethylisoquinolin-2-ium (**21**), 6,7-bis(benzyloxy)-1-ethyl-2-methy- lisoquinolin-2-ium (**22**), 1-(1-(3,4-dimethoxyphenyl)ethyl)-6,7-dimethoxy-2-methylisoquinolin-2-ium (23), 1-ethyl-6,7-dihydroxy-2-methylisoquinolin-2-ium (**24**), 6,7-dimethoxy-3,4-dihydro- isoquinolin-2-ium (**25**), dehydrocavidine (**26**), coptisine (**27**), dehydroapocavidine (**28**) and tetradehydroscoulerine (**29**).

The antimicrobial, antimalarial, cytotoxic, and anti-HIV activities of 26 isoquinolines were studied. The results showed that compound **20** (Figure 4) has antimicrobial effects, compounds **21**, **22** and **23** (Figure 4) have antimalarial effects, compounds **20** and **21** have cytotoxic effects and compounds **24** and **25** (Figure 4) have anti-HIV effects. It is expected that these compounds have the potential to be used as lead compounds for further research and investigation [141].

Berberine alkaloid (**1** in Figure 1) was tested against the oral pathogens *Fusobacterium nucleatum*, *Enterococcus faecalis*, and *Prevotella intermedia*. The MIC values of berberine against these pathogens were 31.25 μg/mL, 3.8 μg/mL and 500 μg/mL, respectively [142].

Sanguinarine (**6** in Figure 1), a benzophenanthridine alkaloid obtained from the root of *Sanguinaria canadensis* L., has shown inhibition activity against methicillin-resistant *Staphylococcus aureus* (MRSA) bacteria, an organism known for its resistance to almost all antibacterial agents. MRSA is responsible for a large number of life-threatening infections. MRSA infections that reach the bloodstream are responsible for numerous complications and fatalities, killing 10–30% of patients. An important predictor of morbidity and mortality in adults is the blood concentrations of vancomycin, the antibiotic of choice to treat this condition [18]. Many of them are skin-related conditions: Skin glands, and mucous membranes. Sanguinarine activity against MRSA strains is in the range of 3.12 to 6.25 µg/mL. Whereas, its MIC values against the two MRSA strains are 3.12 µg/mL and 1.56 µg/mL. Sanguinarine causes lysis of the cell by induction the release of autolytic enzymes [143]. Further, sanguinarine has shown to kill cells and destroy tissues when applied to the skin. The biosynthesis of sanguinarine in plants is via the action of dihydrobenzophe-anthridineoxidase on dihydrosanguinarine.

Dehydrocavidine, coptisine, dehydroapocavidine and tetradehydroscoulerine (**26**–**29,** respectively, in Figure 4) represent the active compounds of Yanhuanglian that extracted from *Corydalis saxicola* Bunting and used in traditional Chinese medicine, and they have shown antibacterial, antiviral and anticancer activities in *in-vivo* studies [144].

**Figure 5:**
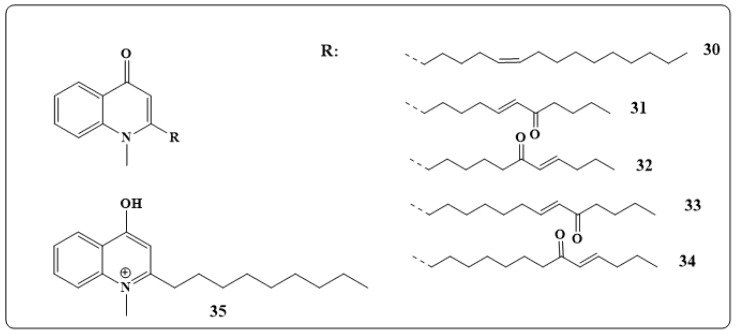
Chemical structures of alkaloids; euocarpines A–E, **30**–**35**, respectively.

Five new quinolone alkaloids, euocarpines A–E (**30**–**35** in Figure 5), were isolated from the fruits of *Evodia ruticarpa* var. *officinalis* (Dode) *C.C.Huang* and were found to exhibit moderate antibacterial activities MIC values of 4–128 μg/mL Compounds with thirteen carbons on side chain showed the highest antibacterial activity with favorable low cytotoxicity [145].

**Figure 6:**
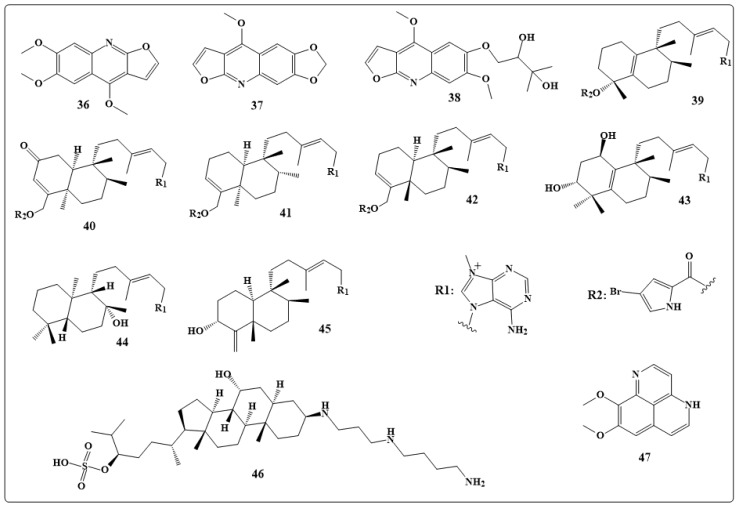
Chemical structures of natural antibacterial alkaloids: Kokusaginine (**36**), maculine (**37**), kolbisine (**38**), agelasines O–U (**39**–**45**), squalamine (**46**) and aptamine (**47**).

The three quinolone alkaloids kokusaginin, maculine, kolbisine (**36**–**38** in Figure 6) were obtained from *Teclea afzelii* Engl.stem bark, and their antimicrobial and antifungal activities were studied. The results revealed that kokusaginine, **36**, was active against Gram-positive and negativebacteria, fungi and *Mycobacterium smegmatis*. While the crude extracts maculine, **37**, and kolbisine, **38**, have resulted in inhibition of 87.5% of the microbes with an MIC value of 19.53 μg/mL. While maculine, **37**, demonstrated a moderate activity against *M. smegmatis* with greater MIC value (156.25 µg/mL) [146].

Eight new quinolone alkaloids were extracted from the *actinomycete Pseudonocardia* sp. CL38489 were found to be potent and selective anti *Helicobacter pylori* agents [147]. Diterpene alkaloids, such as agelasines O–U (**39**–**45** in Figure 6), were obtained from *Okinawan marine sponge Agelas sp*. Studies have shown that agelasines O–R (**39**–**42** in Figure 6) and T (**44** in Figure 6) had antimicrobial activities against several bacteria and fungi [148].

The polyamine alkaloid squalamine (**46** in Figure 6) which was extracted from tissues of the dogfish shark *Squalus acanthiasis* is now considered as a broad-spectrum steroidal antibiotic with potent bactericidal properties against both *Gram-negative* and *Gram-positive* bacteria [149]. 

Aaptamine (**47** in Figure 6) extracted from the Indonesian marine sponge of the genus Xestospongia has shown significant antibacterial activity against *gram-negative bacteria* [150]. Table 2 illustrates the antibacterial activity of the compounds discussed in this section.

### 4.2. Antibacterial Mechanism of Action of Alkaloids

**Figure 7:**
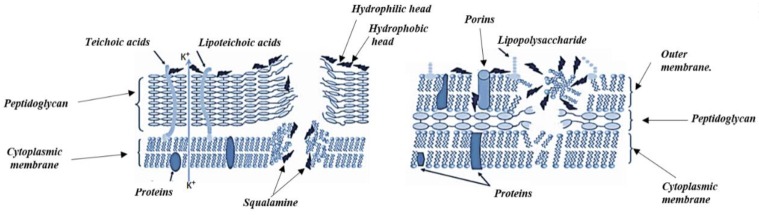
A representation of the mode of action of squalamine antibacterial agent; intracellular ion efflux in *Gram-positive* bacteria caused by the depolarization of the membranes, and membrane disruption in *Gram-negative* bacteria. PG is peptidoglycan, CM is cytoplasmic membrane, and OM is outer membrane.

Most alkaloids are found to be bactericidal rather than being bacteriostatic. For example, squalamine, **42**, was found to be with potent bactericidal properties killing *Gram-positive* and *Gram-negative* pathogens by ≥99.99% in about 1–2 h [151]. The mechanism of action of squalamine, **42**, is illustrated in Figure 7.

The alkaloids mechanism of action as antibacterial agents has been found to be different between each class. In the alkaloids pergularinine and tylophorinidine from the indolizine class, the antibacterial action is due to inhibition of the enzyme dihydrofolate reductase resulting in the inhibition of nucleic acid synthesis [152].

Within the isoquinolone class two mechanisms of bacterial inhibition were shown to occur; Ungereminea phenanthridine isoquinoline exerts its effect through the inhibition of nucleic acid synthesis, while from the studies with benzophenanthridine and protoberberine isoquinolines it was suggested that those agents act by the perturbation of the Z-ring and cell division inhibition, and this was further proved by many studies [97,153,154,155].

On the other hand, synthetic quinolones inhibit the type II topoisomerase enzymes, while the natural quinolone alkaloids lacking the 3-carboxyl function act as respiratory inhibitors by reducing oxygen consumption in the treated bacteria [156,157]. 

Agelasines alkaloids exert their antibacterial activity by the inhibition of the dioxygenase enzyme BCG 3185c, causing a disturbance in the bacterial hemostasis. This result was revealed from overexpression and binding affinity experiments on the anti-mycobacterial alkaloid agelasine D [158]. Saqualamine from the polyamine alkaloid class acts by disturbing bacterial membrane integrity [158,159]. 

## 5. Antiviral Activity

Plants and animals, host a vast number of viruses, often transmitted by insects, such as aphids and bugs. Viral infection resistance can be caused either by biochemical mechanisms that prevent the development and multiplication of the virus or by preventing vectors, such as aphids. The evaluation of the antiviral activity is relatively difficult. Several researchers have studied the effect of alkaloids on viral reproduction. The studies revealed that about 40 alkaloids possess antiviral properties.

**Figure 8:**
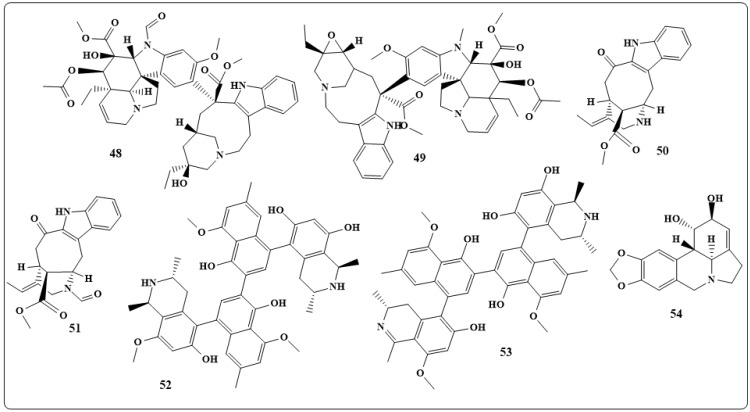
Chemical structures of the antiviral natural alkaloids: Leurocristine (**48**), periformyline (**49**), perivine (**50**), vincaleucoblastine (**51**), michellamines D and F (**52** and **53**) and lycoricidine (**54**).

Leurocristine, periformyline, perivine and vincaleucoblastine (**48**–**51** in Figure 8) are natural alkaloids obtained from *Catharanthus roseus* (L.) and *lanceusPich* (Apocycaceae). Leurocristine, 48, is an active against *mengovirus* extracellular virucidal, poliovirus, *vaccinia,* and *influenza* viruses [160]. Periformyline, **49**, inhibits poliovirus type viruses. Whereas, perivine, **50**, exhibits polio extracellular virucidal activity against *vaccinia,* and vincaleucoblastine, **51**, possesses extracellular virucidal activity against *poliovirus vaccinia and influenza* virus.

The michellamines D and F (**52** and **53** in Figure 8), naphthyl-isoquinoline alkaloids, obtained from the tropical *liana Ancistrocladuskorupensis* have demonstrated HIV-inhibitory [161]. A series of isoquinoline alkaloids as lycorine, lycoricidine (**54** in Figure 8), narciclasine, and cis-dihydronarciclasine, obtained from *Narcissus poeticus* (Amaryllidaceae), have shown significant in vitro activity against *flaviviruses and bunyaviruses*. Poliomyelitis virus inhibition by the above-mentioned compounds occurred at 1 mg/mL [162].

**Figure 9:**
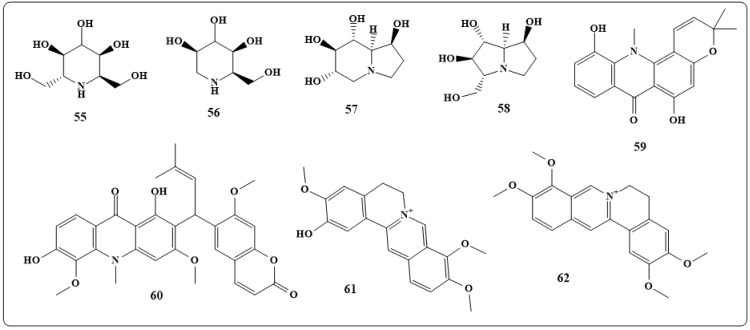
Chemical structures of antiviral natural alkaloid: Homonojirimycin (**55**), 1-deoxymanojirimycin (**56**), castanospermine (**57**), australine (**58**), 5-hydroxynoracronycine (**59**), acrimarine F (**60**), columbamine (**61**), palmitine (**62**).

The alkaloids homonojirimycin (**55** in Figure 9) and 1-deoxymanojirimycin (**56** in Figure 9) were obtained from *Omphaleadiandra* (Euphorbiaceae). Homonojirimycin, **55**, is an inhibitor of several *a*-glucosidases, glucosidase I and glucosidase II. Deoxymanojirimycin, **56**, is an inhibitor of glycoprocessing mannosidase [163]. Castanospermine (**57** in Figure 9) and australine (**58** in Figure 9) are alkaloids present in seeds of *Castanospermum austral* A. Cunn. and *C.* Fraser (Leguminosae) and reduce the ability of HIV to infect cultured cells and have the potential for treating AIDS [164]. Sesquiterpene alkaloids obtained from *Tripterygium hypoglaucum* (H. Lév.) and *Tripterygium wilfordii* Hook. f. (Celastraceae) were found to possess anti-HIV activity [165].

The acridone alkaloids, 5-hydroxynoracronycine—same compound of 11-hydroxynorac- ronycine (**59** in Figure 9) and Acrimarine F (**60** in Figure 9) were extracted from *Citrus alata* (Tanaka) plants and were found to be very effectiveagainst Epstein-Barr virus [166,167,168]. Columbamine, palmitine(**61, 62** in Figure 9), and berberine are alkaloids with potent activity against HIV-1 and can be found in many plants, includingAnnonaceae (Coelocline), *Berberis vulgaris* (Berberidaceae), Menispermaceae and Papaveraceae [169,170,171]. A concise summary of the antiviral activities of alkaloids 48–62 is depicted in Table 3.

## 6. Antifungal Activity

The spread of resistance to antifungal agents has accelerated in the recent few years. The rate of morbidity and mortality has enhanced, due to resistance to antifungal agents. Since the molecular processes in humans and fungi are similar, there is always a risk that the fungal cytotoxic substance is toxic to the host cells. Consequently, patients with a reduced immune system, such as those with transplants, cancer and diabetics who do not respond appropriately to the current medications have ignited the need for new antifungal medicines [93]. The current antifungal drugs suffer from many side effects as irritation, diarrhea, and vomiting; furthermore, it is less effective, due to the development of resistance to those drugs by the diverse available fungi. The potency and effectiveness of the antifungal medicines developed during 1980–1995, such as the imidazoles and triazoles, which inhibit the fungal cell processes have gradually decreased, due to the development of resistance by the microorganism [172]. Therefore, researchers are looking for new potent entities to replace the currently used antifungal agents. A respected number of biologically active compounds were isolated from medicinal plants and are widely used as mixtures or pure compounds to cure a variety of diseases. It is estimated that about 250,000 to 500,000 species of plants are growing on our planet. However, humans are using only 1 to 10% of those plants [173]. This treasure should be utilized to isolate and develop new antifungal agents by using new methods and techniques [174]. For example, chemical entities isolated from plants, such as the indole derivatives, dimethyl pyrrole and hydroxydihydrocornin-aglycones have shown promising antifungal activities [174]. However, pure medicines derived from these chemical entities still to be developed. 

It should be emphasized that the annual mortality rate, due to fungi was constant through several decades, and the resistance to antifungal drugs has emerged only in the recent two to three decades [175,176,177]. Thus, a combined effort from chemists, biologists, pharmacologists and etc. is crucially needed to combat the issue of microorganisms’ resistance to drugs. Consequently, great emphasis has been placed on developing a more detailed understanding of antimicrobial resistance mechanisms, improved methods for detecting resistance when they occur, new antimicrobial options for treating infections caused by resistant organisms, and ways to prevent the emergence and spread of resistance in the first place [174].

In this section of the review, we attempted to report on some important antifungal compounds obtained from plants.

### Antifungal Activity of Alkaloids 

**Figure 10:**
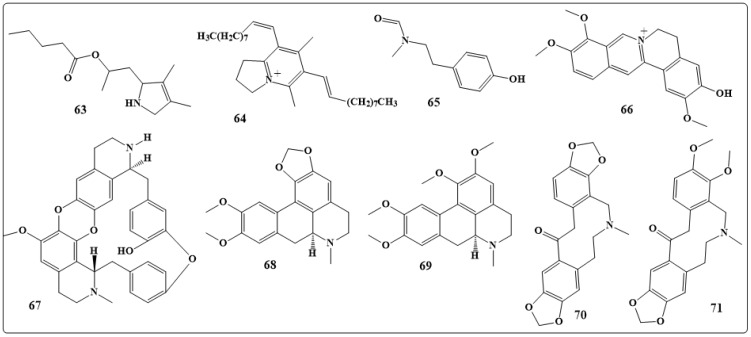
Chemical structures of antifungal natural alkaloids: 2-(3,4-dimethyl-2,5-dihydro-1H-pyrrol-2-yl)-1-methylethyl pentanoate (**63**), 6,8-didec-(1Z)-enyl-5,7-dimethyl-2,3-dihydro-1H-indolizinium (**64**), N-methyl-N-formyl-4-hydroxy-beta-phenylethylamine (**65**), Jatrorrhizine (**66**), (+)-Cocsoline (**67**), dicentrine (**68**), glaucine (**69**), protopine (**70**) and alpha-allocryptopin (**71**).

Extraction of the opium poppy *Papaver somniferum* L. has resulted in the isolation of morphine which is considered the first member [156]. Recently, a new alkaloid, 2-(3,4-dimethyl-2,5-dihydro-1H-pyrrol-2-yl)- 1-methylethyl pentanoate (**63** in Figure 10) was isolated from the plant *Datura metel* var. *fastuosa* (L.) *Saff.,* and has demonstrated activity against *Aspergillus* and *Candida* species when tested both in vitro and in vivo [178,179]. 

Another alkaloid, 6,8-didec-(1Z)-enyl-5,7-dimethyl-2,3-dihydro-1H-indolizinium (**64** in Figure 10) was isolated from *Anibapanurensis* and demonstrated excellent activity against the strain of *C. albicans* [180]. 

β-Carboline, a tryptamine and twophenylethylamine-derived alkaloids along with N-methyl-N-formyl-4-hydroxy-beta-phenylethylamine (**65** in Figure 10) from *Cyathobasisfruticulosa* [20] and *haloxylines A* and *B*, new piperidine from *Haloxylon schmittianum* Pomel have shown a potential antifungal activity [181].

Jatrorrhizine (**66** in Figure 10) isolated from *Mahonia aquifolium (Pursh)* Nutt. has shown the most potent antifungal inhibitory in all studied fungi with MIC between 62.5 to 125 μg/mL, while the crude extract, berberine, and palmatine showed reduced inhibitory activity with MIC of 500 to >/= 1000 μg/mL [182].

(+)-Cocsoline (**67** in Figure 10) is a bisbenzylisoquinoline alkaloid isolated from *Epinetrumvillosum* has demonstrated a good antifungal activity [183]. The alkaloids N-methylhydrasteinehydroxylactam and 1-methoxyberberine chloride isolated from *Corydalis longipes* D. Don demonstrated great inhibitory activity [184]. Four alkaloids, dicentrine (**68** in Figure 10), glaucine (**69** in Figure 10), protopine (**70** in Figure 10), and alpha-allocryptopin (**71** in Figure 10) were isolated from *Glaucium oxylobum* Boiss and Buhse have exhibited good activity against *Microsporumgypseum, Microsporumcanis, T. mentagrophytes* and *Epidermophytonfloccosum* [185].

**Figure 11:**
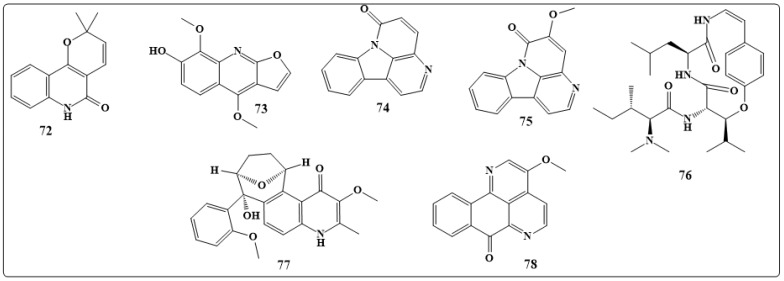
Chemical structures of antifungal natural alkaloids: Flindersine (**72**), haplopine (**73**), canthin-6-one (**74**), 5-methoxy-canthin-6-one (**75**), frangulanine (**76**), waltherione A (**77**) and 3-methoxisampangin (**78**).

Flindersine (**72** in Figure 11) and haplopine (**73** in Figure 11) obtained from *Haplophyllum sieversii* Fisch. were growth-inhibitory compounds against various fungi [186]. Canthin-6-one (**74** in Figure 11) and 5-methoxy-canthin-6-one (**75** in Figure 11) of *Zanthoxylumchiloperone* var. *angustifolium* exhibited antifungal activity against *C. albicans,* A. *fumigatus* and T. *mentagrophytes* [187]. Frangulanine (**76** in Figure 11), a cyclic peptide alkaloid and waltherione A (**77** in Figure 11), quinolinone alkaloids from leaves of *Melochiaodorata* were reported to exhibit antifungal activities against a broad spectrum of pathogenic fungi [188].

Furthermore, anodic alkali aninolinate has demonstrated inhibitory activity against all 10 fungi tested and demonstrated a particularly high sensitivity to this compound, showing germination levels of less than 10% [189]. From the root of *Dictamnusdasycarpus* two antifungal fructoxin alkaloids were isolated. 3-Methoxisampangin (**78** in Figure 11) of *cleistopholispatens* showed vast inhibitory activity against each of *C. albicans,* A. *fumigatus,* and *C. neoformans* [190]. Table 4 lists the antifungal activity of the compounds discussed in this section.

## 7. Toxicity of Plant Secondary Metabolites

Through hundreds of years, people have been used plant extracts for the treatment of a variety of diseases, such as snakebite, fever and insanity. However, a number of plants containing alkaloids are classified as main plant toxins because of their vast structural diversity and different mechanism of actions. Inhalation or swallowing toxic alkaloids by humans or animals might result in a certain mechanism involving transporters, enzymes and receptors at certain cells and tissues, and therefore, causing musculoskeletal deformities and hepatotoxic effects. These toxic alkaloids include tropane, piperidine, pyrrolizidine and indolizidine. The most adverse effects of these entities are vomiting, mild gastrointestinal perturbation, teratogenicity, arrhythmias, itching, nausea, psychosis, paralysis, and death [188].

## 8. Conclusions

Secondary metabolites isolated from different plants have proven to be useful for humans and animals alike. Studies on a number of these alkaloids have demonstrated that a vast number of them possess many pharmacological activities. These activities include anticancer, antibacterial, antiviral, antioxidant and antifungal. However, when high doses of the secondary metabolites are consumed, toxic effects may be observed and in sometimes fatalities are imminent. For instance, some alkaloids have proven to lead to paralysis, asphyxia, and in sometimes to death. During the past few years researches have been conducted to make new derivatives of the isolated secondary metabolites aiming to obtain medicines with more potency, less toxicity, and more resistant to different microorganisms. It is hoped that the state-of-the-art methods and sophisticated techniques along with computational methods, will make the path for generating novel potent drugs, based on natural products, be shorter and beneficial.

Several plants contain secondary metabolites which are toxic and may cause danger to humans when administered. However, the cases in which fatal plant poisonings occur are negligible. The most common incidents of toxicity are those involving the abuse of plants for hallucinogenic purposes. Utilizing toxicological analysis of such secondary metabolites may help in the diagnosis of poisoning or abuse cases. Among the toxic secondary metabolites are harmaline, ibogaine, kawain, cytisine, dimethyltryptamine, harmine, aconitine, atropine, coniine, colchicine, taxine, mescaline, and scopolamine, which are often involved in fatal poisonings [190].

## Figures and Tables

**Table 1:** The anticancer compounds discussed in this section and their activities.

Alkaloids	Anticancer Activity
Berberine Matrine	Inhibits the proliferation of breast, lung, colon and liver cancer cell lines Inducing the cell cycle arrest or apoptosis in cancer cell Inhibits the proliferation of cancer cell by G1 cell cycle arrest or apoptosis
Piplartine	Antitumor related to its antiproliferative effect
Piperine Sanguinarine Tetrandrine	Antitumor and immunomodulatory Induces cell cycle arrest at different phases or apoptosis in a variety of cancer cell Induces different phases of cell cycle arrest depends on cancer cell types
Aporphine	Antitumor activity against small cell, lung cancer and breast cancer cells, in addition to a high antitumor activity against epidermoid carcinoma
Apomorphine	Antioxidant and antipoliferative activities
Harmine, harmaline harmalacidine, vasicinone	Inhibit topoisomerase 1 resulting in antiproliferation of cancer cells
Evodiamine Sanguinarine	Induce cell cycle arrest or apoptosis, inhibiting the angiogenesis, invasion, and metastasis in a variety of cancer cell lines
Matrine	Inhibits the proliferation of various types of cancer cells mainly through the mediation of G1 cell cycle arrest or apoptosis
Tetrandrine	Induces apoptosis in many human cancer cells, including leukemia, bladder, colon, hepatoma, and lung

**Table 2:** Antibacterial compounds, discussed in this section, and their antibacterial activity.

Alkaloids	Antibacterial Activity
Clausenol, Kokusaginine, Maculine, Kolbisine, squalamine, Aaptamine	Active against *Gram-positive* and *negative* bacteria and fungi
*R-* Mahanine	Antimicrobial activity against *Bacillus cereus* and *Staphylococcus aureus*
hapalindole X, deschlorohapalin-dole I, 13-hydroxy dechlorofonto-namide, hapalonamide H	Potent activity against both *Mycobacterium tuberculosis* and *Candida albicans*
Indolizdine	Antibacterial activities against *Cryptococcus neoformans*, *Aspergillus fumigatus*, methicillin-resistant *Staphylococcus aureus* and *Mycobacterium* intracellular
Isoquinolines	Antimalarial, cytotoxic, and anti-HIV effects.
Sanguinarine,	Antibacterial activity against methicillin-resistant *Staphylococcus aureus*

**Table 3:** Antiviral compounds, discussed in this section, and their antiviral activity.

Alkaloids	Antiviral Activity
Leurocristine	Active against *mengovirus* extracellular virucidal, poliovirus, *vaccinia*, and *influenza* viruses
Periformyline	Inhibits poliovirus type viruses
Perivine	Exhibits polio extracellular virucidal activity against *vaccinia*
Vincaleucoblastine	Possesses extracellular virucidal activity against poliovirus *vaccinia* and *influenza* virus.
Michellamines D and F	HIV-inhibitory
Homonojirimycin	Inhibitor of several a-glucosidases
Deoxymanojirimycin	Inhibitor of glycoprocessing mannosidase
Castanospermine, australine	Reduce the ability of the human immunodeficiency virus (HIV) to infect cultured cells, and have potential for treating AIDS
Sesquiterpene	Anti-HIV activity
5-hydroxynoracronycine, Acrimarine F	Remarkable inhibitory effects on *Epstein-Barr* virus activation
Columbamine, Berberine, Palmitine	Inhibitors alkaloids against HIV-1

**Table 4:** Antifungal compounds, discussed in this section, and their antifungal activity.

Alkaloids	Antifungal Activity
2-(3,4-dimethyl-2,5-dihydro-1H-pyrrol-2-yl)-1-methylethyl pentanoate	Activity against *Aspergillus* and *Candida* species
6,8-didec-(1Z)-enyl-5,7-dimethyl-2,3-dihydro-1H-indolizinium	Good activity against a drug-resistant strain of *C. albicans*
β-carboline, Cocsoline	Effective against all fungal species and marginal activity
Dicentrine, glaucine, Protopine, alpha-allocryptopin	Good activity against *Microsporumgypseum*, *Microsporumcanis*, *T. mentagrophytes* and *Epidermophytonfloccosum*
Canthin-6-one, 5-methoxy-canthin-6-one	Antifungal activity against *C. albicans*, *A. fumigatus* and *T. mentagrophytes*
Frangulanine, Waltherione, quinolinone alkaloids	Exhibit antifungal activities against a broad spectrum of pathogenic fungi
3-methoxisampangin	Significant antifungal activity against *C. albicans*, *A. fumigatus*, and *C. neoformans*
